# *NAV3* Missense Variant in a Homozygous State: Strengthening Links to Neurodevelopmental Disorder

**DOI:** 10.2174/0109298673367919250626005640

**Published:** 2025-07-24

**Authors:** Muhammad Umair, Anwar Ullah, Najumuddin, Gohar Zaman, Ishtiaq Ahmed, Fazl Ullah, Muhammad Bilal, Majid Alfadhel

**Affiliations:** 1 Medical Genomics Research Department, King Abdullah International Medical Research Center (KAIMRC), King Saud Bin Abdulaziz University for Health Sciences (KSAU-HS), Ministry of National Guard Health Affairs (MNGH), Riyadh, Saudi Arabia;; 2 Institute of Paramedical Sciences Khyber Medical University, Peshawar, Pakistan;; 3 Department of Applied Sciences, Hamdard University, Karachi, Pakistan;; 4 Department of Computer Science, Abbottabad University of Science and Technology, Havelian, Abbottabad, Pakistan;; 5 Institute of Biochemistry and Biotechnology, Pir Mehr Ali Shah Arid Agriculture University Rawalpindi, 46000, Pakistan;; 6 Metropole Laboratories (Private) Limited, Islamabad, 44000, Pakistan;; 7 Lady Reading Hospital Peshawar, Peshawar, Khyber Pakhtunkhwa, 25000, Pakistan;; 8 Department of Pathology and Laboratory Medicine, Aga Khan University, Karachi, Pakistan;; 9 Genetic and Precision Medicine Department, King Abdullah Specialized Children Hospital, King Abdulaziz Medical City, Ministry of National Guard Health Affairs (MNGHA), Riyadh, 11426, Saudi Arabia

**Keywords:** Neurodevelopmental disease, WES, *NAV3*, missense mutation, homozygous, autosomal recessive

## Abstract

**Introduction:**

Neurodevelopmental disorders (NDDs) represent a diverse and heterogeneous group of conditions, including global developmental delay (GDD), autism spectrum disorder (ASD), and neurodevelopmental encephalopathy with epilepsy (NDEE). While these disorders often share phenotypic similarities, their underlying genetic causes can vary widely, making clinical diagnosis challenging.

**Methods:**

In this study, we performed whole-genome sequencing (WGS) on a family having an autosomal recessive neurodevelopmental disorder. The proband (II-2) underwent WGS, followed by variant filtering through an in-house bioinformatics pipeline. Sanger sequencing and 3D protein modeling were performed to confirm the pathogenicity of the identified variant.

**Results:**

A novel biallelic missense variant in the *NAV3* (c.3430T>C; p.Ser1144Pro) was detected using WGS and Sanger sequencing. Subsequently, 3D protein modeling revealed significant alterations in the secondary structure of NAV3, indicating a potential pathogenic effect.

**Discussion:**

The identification of a novel biallelic missense variant in *NAV3* adds a new layer to our understanding of its potential contribution to autosomal recessive neurodevelopmental disorders. This case expands the mutational landscape of *NAV3* and underscores its emerging significance in neurodevelopment.

**Conclusion:**

This study reports a novel *NAV3* variant in association with autosomal recessive NDD, contributing to the growing body of evidence supporting the involvement of *NAV3* in human neurodevelopment. Functional validation and identification of additional patients will be essential to establish definitive genotype-phenotype correlations and uncover the mechanistic pathways underlying NAV3-associated disorders.

## 1 INTRODUCTION

Neurodevelopmental disorders (NDDs) represent a heterogeneous group of rare conditions characterized by delays and impairments in cognitive, emotional, and motor development [1]. The global burden of NDDs has significantly increased, affecting a large number of children worldwide [2]. These disorders arise from disruptions in tightly regulated processes essential for brain development. The etiology of NDDs is highly diverse, contributing to a spectrum of clinical manifestations, including cognitive deficits, impaired communication, psychomotor dysfunction, and challenges in adaptive behavior. NDDs encompass a range of disorders such as autism spectrum disorder (ASD), intellectual disability (ID), epilepsy, and attention deficit hyperactivity disorder (ADHD). The shared clinical features observed across these conditions suggest the involvement of common molecular pathways, highlighting the need for further investigation into their underlying mechanisms [3].

Elucidating the genetic architecture of NDDs has led to the identification of various types of mutations across different genes. These mutations include chromosomal rearrangements, single-nucleotide polymorphisms (SNPs), copy number variations (CNVs), missense mutations, frameshift mutations, and small insertions/deletions [3-5].

Despite the complexity and clinical overlap among NDDs, identifying the underlying genetic mutations remains essential to addressing the heterogeneity of these disorders. Precise detection of the causative gene or mutation is crucial for confirming diagnoses and guiding personalized management strategies [6, 7].

Unraveling the genetic basis of NDDs is pivotal for understanding the molecular pathology, enabling better genotype-phenotype correlations, and facilitating targeted therapeutic approaches. Investigating familial cases provides valuable insights into inheritance patterns and pathogenic variants, contributing to the resolution of this diagnostic challenge. Additionally, large-scale case-control studies involving individual patients and healthy controls are essential for identifying potential risk loci or variations, although such studies may not always pinpoint definitive causative genes or mutations [8].

The advent of next-generation sequencing technologies, including whole-genome sequencing (WGS), whole-exome sequencing (WES), targeted sequencing, and RNA profiling, has revolutionized the ability to achieve rapid and accurate genetic diagnoses, facilitating the identification of disease-causing variants in complex disorders [9].

Recently, *NAV3* (OMIM: 611629) has emerged as a novel candidate gene implicated in rare neurodevelopmental disorders, also known as Umair-Alfadhel Neurodevelopmental Disorder (OMIM: 621182), as demonstrated by two independent studies [3, 4]. Ghaffar *et al.* [3] expanded the understanding of NAV3's role by identifying *NAV3* variants in eleven individuals presenting with intellectual disability, microcephaly, and neuronal growth abnormalities. Functional studies in human cell lines and zebrafish models demonstrated NAV3's critical involvement in microtubule stabilization and neuronal development, highlighting its essential role in maintaining neuronal architecture.

In a separate study by Umair *et al.* [4], a bi-allelic frameshift variant in *NAV3* was identified in a Saudi proband with a complex neurodevelopmental disorder. Functional analyses, including 3D protein modeling and RT-qPCR, revealed a significant reduction in *NAV3* expression and structural protein disruption, further supporting its pathogenic role.

Collectively, these studies position *NAV3* as a key player in neurodevelopmental processes, where its dysfunction leads to severe cognitive, structural, and neuromuscular impairments. These findings underscore the importance of *NAV3* in brain development and pave the way for future studies to explore its potential as a diagnostic biomarker and therapeutic target.

Familial NDDs provide an invaluable model for investigating the interplay between genetic and non-genetic factors contributing to the pathophysiology of these disorders, particularly in the context of a shared genetic background. Extensive research has focused on pedigrees with multiple affected offspring exhibiting incomplete penetrance and phenotypic heterogeneity, as well as on monozygotic twins presenting with discordant phenotypes [10].

Identifying the genetic cause of these disorders is essential for understanding the underlying molecular mechanisms, improving genotype-phenotype correlations, and guiding clinical management. Whole-genome sequencing (WGS) has proven to be a powerful tool in this context. In the present study, WGS was performed on a family with a complex neurodevelopmental disorder, leading to the identification of a novel mutation in the *NAV3* gene, further expanding its role in neurodevelopmental disease pathology.

## 2 SUBJECTS AND METHODS

### 2.1 Ethical Approvals and DNA Extraction

The current study was conducted under the Declaration of Helsinki guidelines and approved by the Research Ethics Committee of KAIMRC) [NRC23R-177-02; RC-IRB87-23]. Informed written consent was obtained from the legal guardians, including permission to publish identifiable data.

A family with two affected members was enrolled in this study. 5 mL of venous blood was collected from each participant and stored under appropriate conditions. Genomic DNA was subsequently extracted and quantified using standard protocols [11, 12].

### 2.2 Whole Genome Sequencing (WGS)

Whole genome sequencing (WGS) was performed on the index case (II-2) using the TruSeq Nano DNA protocol and the Illumina HiSeq X Ten platform [13]. Raw sequencing reads were aligned to the GRCh38 reference genome using BWA 0.6.2, and duplicate reads were marked and removed with Picard. Single-nucleotide variants (SNVs) were identified using SAMtools, while short insertions and ANNOVAR.

For quality control, variants with a read depth below 10× or a quality score below 20 were excluded. Common variants with a minor allele frequency (MAF) > 0.01 were filtered out, retaining only rare variants (MAF < 0.01) for further analysis. Public databases, including dbSNP, 1000 Genomes, and NHLBI 6500, alongside over 2000 in-house genomes/exomes, were used as controls for variant filtering [14].

### 2.3 *In-silico* Prediction for Variant Pathogenicity

Multiple *in silico* prediction tools were employed to evaluate the potential functional impact of the identified variants. These included SIFT, MetaSVM, HDIV, PolyPhen2, CADD, LRT, MutationAssessor, MutationTaster, FATHMM, MetaLR (0.3076: VUS), VEST, GERP++, fitCons, PhyloP, DANN, and SiPhy. To further classify the pathogenicity of the identified mutation, the Program for Personalized and Genomic Medicine online tool was utilized. Additionally, DECIPHER was used to support the variant's clinical relevance [15-31] (Tables **1** and **2**). For further variant prioritization, we focused on those that met the following criteria: (a) Located in genes highly expressed in the brain, (b) Previously implicated in neurological disorders, (c) residing in highly conserved regions across species, and (d) involving genes already associated with neurodevelopmental disorders (NDDs).

### 2.4 Sanger Sequencing

The prioritized variants were validated through bi-directional Sanger sequencing, and segregation analysis was performed across all available family members, following established protocols [32]. Primers flanking the candidate variant regions were designed using Primer3 software (version 0.4.0) to ensure accurate and specific amplification of the targeted DNA sequences.

### 2.5 NAV3-3D Structure Prediction and Conservation Analysis

The 3D structure of NAV3^WT^`^(121-1200aa)^, was predicted using the I-TASSER. The structure was subjected to homology modelling, and the 3D structure of NAV3^SER1144PRO^ was predicted using MODELLER software. Using AMBER ff99SB by using UCSF Chimera 1.8.1, the mutated structure was subjected to energy minimization. Rotamers and amino acid outliers were removed through WinCoot 0.9.8.1. The predicted structure quality was checked using VERIFY3D and MolProbity. Finally, Pymol and USCF Chimera 1.8.1 were used to optimize the structure for mutational and structural analysis [33]. Multiple sequence alignment was conducted using Clustal Omega and visualized with Jalview.

## 3 RESULTS

### 3.1 Clinical Presentation of the Family

A five-member pedigree of the family is presented in Fig. (**1A**), with the pattern of inheritance suggesting an autosomal recessive mode. The two affected siblings-II-2 (age 10) and II-3 (age 14)-exhibited developmental delay, intellectual disability, walking difficulty, and impaired learning and speech. Both individuals also experienced recurrent febrile seizures.

Speech assessments revealed ambiguous articulation, raising concerns about vocal cord involvement. To investigate this, flexible laryngoscopy was performed, confirming vocal fold hypomobility. Additionally, vocal electromyography (EMG) demonstrated an abnormal pattern, characterized by reduced motor unit action potentials (MUAPs) and decreased interference patterns (IPs)-indicative of neuromuscular dysfunction affecting vocal fold movement.

The affected individuals exhibited dysmorphic features, including right microphthalmia, prominent anteriorly displaced ears, micrognathia (small chin), high-arched palate, and a broad nasal bridge with a bulbous nose.

For individual II-2, growth parameters included: height of 127.6 cm (4^th^ percentile, -1.71 SD), weight of 28.2 kg (19^th^ percentile, -0.89 SD), and head circumference of 51.3 cm (12^th^ percentile, -1.17 SD).

For individual II-3, growth measurements showed height of 131.5 cm (<1st percentile, -3.89 SD), weight of 34.5 kg (1st percentile, -2.20 SD), and head circumference of 51.6 cm (2^nd^ percentile, -2.01 SD).

Both affected siblings demonstrated early childhood developmental delays, including delayed sitting, crawling, walking, and speech acquisition. Currently, they communicate in one- to two-word sentences with poor articulation and non-fluent speech. Notably, no abnormalities were detected in vision, hearing, or sleep patterns (Table **1**).

### 3.2 Brain MRI

Brain MRI revealed generalized cerebellar atrophy, predominantly affecting the ventricles and cerebellar folia, with a small vermis. No evidence of hydrocephalus or space-occupying lesions was observed.

Supratentorial imaging showed areas of high signal intensity in the peritrigonal region, most prominently on FLAIR sequences, likely representing the terminal zones of myelination-though myelination appeared age-appropriate.

The gray-white matter differentiation and midline structures were preserved, with no midline shift, mass effect, or hemorrhage detected. A gross assessment of the orbits and paranasal sinuses was unremarkable, and no abnormal diffusion restriction was identified. Additionally, the bony calvarium demonstrated normal signal intensity, with no focal lesions (Fig. **1B**).

### 3.3 Genomic Findings

Whole-genome sequencing (WGS) identified approximately 4.1 million unique variants, comprising 3.5 million single-nucleotide variants (SNVs) and 0.6 million insertion/deletions (Indels). Rare variants with minor allele frequency (MAF) <0.01 (~0.7 million) and novel variants were prioritized for downstream analysis. Missense variants were selected based on the predefined prioritization criteria outlined earlier [13, 14]. Segregation analysis using Sanger sequencing confirmed a homozygous missense variant (c.3430T>C; p.Ser1144Pro) in the *NAV3* gene (MIM: 611629, Transcript: ENST00000397909.7 / NM_001024383.2) in both affected siblings (Figs. **1A**, **C**; Table **2**).

*NAV3* has only recently emerged as a candidate gene in neurodevelopmental disorders (NDDs), with limited variants reported to date (Table **3**). Multiple sequence alignment was performed with Clustal Omega depicted that the mutation affects the highly conserved region among the species (Fig. **1D**).

### 3.4 NAV3 Impact on Protein Structure and Function

The predicted wild type structure of *NAV3* had a C-score was -0.09, with estimated TM-Score = 0.70±0.12 and Estimated RMSD = 9.3±4.6Å. The 3D-1D score of NAV3^WT^ and NAV3^SER1144PRO^ was >=0.2. NAV3^WT^ contains 47 α-helices and 4 β-sheets. SER1144PRO resided in α45. Due to the induction of mutation in NAV3^SER1144PRO^, the α13 converted into a loop, there was induction of α-helix between α18 and β3, and α22 converted into a loop, as compared to NAV3^WT^ (Fig. **1E**).

### 3.5 Ser1144Pro Change

The p.Ser1144Pro substitution introduces a significant structural perturbation due to proline's unique biochemical properties. Proline is widely recognized as a “helix breaker” because of its rigid, cyclic pyrrolidine ring, which restricts φ (phi) torsion angles and disrupts the regular hydrogen bonding network essential for maintaining α-helices and β-sheets [34]. In α-helices, proline insertion commonly induces a kink or bend, leading to destabilization or even complete disruption of the helical conformation. Likewise, β-sheets require an extended backbone configuration, which proline's restricted structure cannot accommodate, resulting in misalignment and structural distortion [35].

Conversely, proline is frequently enriched in turns and loops, where its ability to enforce sharp bends enhances structural flexibility. However, the replacement of serine — a smaller, more flexible residue — with proline markedly reduces local flexibility, imparting increased rigidity to the protein backbone. This shift in regional dynamics can critically affect protein stability, folding efficiency, and functional interactions.

Pathogenicity prediction tools, including SIFT, PolyPhen-2, CADD, MutationTaster, and others, consistently classified the c.3430T>C (p.Ser1144Pro) variant as disease-causing (Table **4**), supporting its deleterious impact on *NAV3* protein function and its involvement in neurodevelopmental disorder pathology. Furthermore, the WGS data analysis pipeline employed in this study is summarized in Fig. (**2**).

## 4 DISCUSSION

In this study, we report the molecular and clinical characterization of a five-member consanguineous family with two affected siblings (II-2, age 10; II-3, age 14) presenting with complex neurodevelopmental phenotypes, including developmental delay, intellectual disability, motor difficulties, speech impairment, and dysmorphic features. Additionally, the younger sibling (II-2) exhibited febrile seizures and ambiguous speech, indicative of vocal cord involvement, which was further supported by laryngoscopic examination and vocal EMG, revealing vocal fold hypomobility and abnormal neuromuscular patterns.

Whole genome sequencing (WGS), followed by Sanger sequencing validation, identified a homozygous missense variant (c.3430T>C; p.Ser1144Pro) in the *NAV3* gene (MIM: 611629). This variant resides in the microtubule-binding domain (MTBD) a highly conserved region critical for NAV3’s role in microtubule stabilization and neuronal development.

The clinical features observed in our two affected individuals (II-2 and II-3) show notable similarities to previously reported cases harboring *NAV3* variants. Both individuals exhibited developmental delay, intellectual disability, and difficulty with walking, learning, and speech — a constellation of features aligning with phenotypes described in several NAV3-associated neurodevelopmental disorders (NDDs).

Our findings parallel those reported by Ghafar *et al.* [3], particularly families PKMR471, PE30, and PK1, where affected individuals presented with global developmental delay and speech impairment. However, a distinctive feature in our cohort is ambiguous speech linked to vocal cord involvement, confirmed through flexible laryngoscopy and vocal EMG — findings not widely described in previous NAV3-related cases, suggesting a novel aspect of the phenotype.

Moreover, dysmorphic features were observed in both affected siblings, characterized by microphthalmia, anteriorly displaced ears, broad nasal bridge, and small chin, overlapping with the craniofacial dysmorphism documented in cases PKMR471 and PED4263, which included dentofacial abnormalities and hypertelorism [3]. This further supports NAV3’s potential role in craniofacial development. Notably, febrile seizures in our index patient (II-2) stand out as an atypical feature not prominently reported in the comparative cases, raising the possibility that *NAV3* variants might contribute to seizure susceptibility.

The 12q21.2 region harbors the *NAV3* gene, which encodes a cytoskeletal-associated protein consisting of 2,385 amino acids. To date, four homozygous and four heterozygous pathogenic variants have been reported in unrelated families affected by neurodevelopmental disorders (NDDs). Additionally, an ExWAS study on an autism spectrum disorder (ASD) cohort identified 12 frameshift, nine nonsense, and three splice-site de novo variants (Table **3**), further supporting the gene’s role in neurodevelopment.

Kakar *et al.* [36], recently identified three novel variants in *NAV3* among three unrelated Pakistani families with NDDs [36], adding to the growing evidence of NAV3’s pathogenic involvement across diverse populations. The presence of *NAV3* mutations in NDD cases from varied ethnic backgrounds reinforces the clinical significance of our identified homozygous missense mutation (c.3430T>C; p.Ser1144Pro). Moreover, functional studies of *NAV3* have highlighted its critical role in brain physiology, suggesting that mutations in this gene may disrupt cytoskeletal dynamics, contributing to neurological dysfunction. This aligns with our findings and supports NAV3’s growing recognition as a key player in neurodevelopmental pathways.

The *NAV3* protein plays a crucial role in organogenesis, particularly within the nervous system, where it supports neuronal guidance and directs axonal growth [37]. Several alternatively spliced transcript variants of *NAV3* have been identified, though only one has been confirmed as full-length. Functionally, *NAV3* parallels the Caenorhabditis elegans gene unc-53, which regulates axon guidance [38].

NAV3 expression is notably enriched in the neuronal nuclear membranes across key brain regions, including the cerebellum, cerebral cortex, midbrain, and hippocampus. Additionally, its expression increases in reactive astrocytes following brain injury [39]. Postmortem analyses of patients with Alzheimer's disease (AD), Parkinson's disease (PD), and amyotrophic lateral sclerosis (ALS) reveal *NAV3* expression in the cytoplasm, axons, and dendrites of the frontal cortex [40]. Furthermore, *in vivo* studies in zebrafish demonstrated that loss-of-function and gain-of-function mutations in *NAV3* disrupt organogenesis, supporting its essential role in development [41]. Given its crucial role in neurodevelopment and disease pathology, early detection of pathogenic *NAV3* variants is vital for prevention strategies. Advances in reproductive technologies, such as preimplantation genetic testing for monogenic disorders (PGTM) and aneuploidy (PGTA), offer potential avenues for reducing the transmission of pathogenic variants in families with a known genetic predisposition [42, 43]. Additionally, genetic counseling combined with carrier screening can further aid in identifying at-risk individuals and guiding informed reproductive decisions to prevent the recurrence of NAV3-associated disorders.

Our study contributes valuable insights into NAV3’s involvement in neurodevelopmental disorders (NDDs). However, a key limitation is the small sample size, which limits generalizability. Future research should integrate *in vitro* and *in vivo* models to functionally characterize *NAV3* mutations and investigate downstream effects. Additionally, larger, more diverse cohorts are essential to define the clinical spectrum and prevalence of NAV3-related NDDs. Given NAV3’s emerging role in neurodevelopment, we propose further investigations into its molecular mechanisms and pathophysiological contributions.

## CONCLUSION

Using whole genome sequencing (WGS), segregation analysis, and 3D protein modeling, our findings suggest that the *NAV3* mutation (c.3430T>C; p.Ser1144Pro) is a causative genetic factor underlying neurodevelopmental disease in the investigated family. This mutation, located within a highly conserved and functionally critical domain of NAV3, likely disrupts protein stability and neuronal function, contributing to the observed clinical phenotype. 

Our study underscores the translational and diagnostic potential of the *NAV3* (c.3430T>C; p.Ser1144Pro) mutation in neurodevelopmental disorders. Integrating *NAV3* into genetic screening panels could facilitate early diagnosis, personalized clinical management, and genetic counseling for affected families. Moreover, future functional studies are crucial to unravel NAV3’s precise molecular roles, potentially unveiling therapeutic targets and paving the way for novel treatment strategies to improve patient outcomes [44].

## AUTHORS' CONTRIBUTIONS

The authors confirm their contribution to the paper as follows: study conception and design: MU; data analysis or interpretation: N., GZ, MB; methodology: IA: investigation: FU; draft manuscript: AU, MA. All authors reviewed the results and approved the final version of the manuscript.

## Figures and Tables

**Fig. (1):**
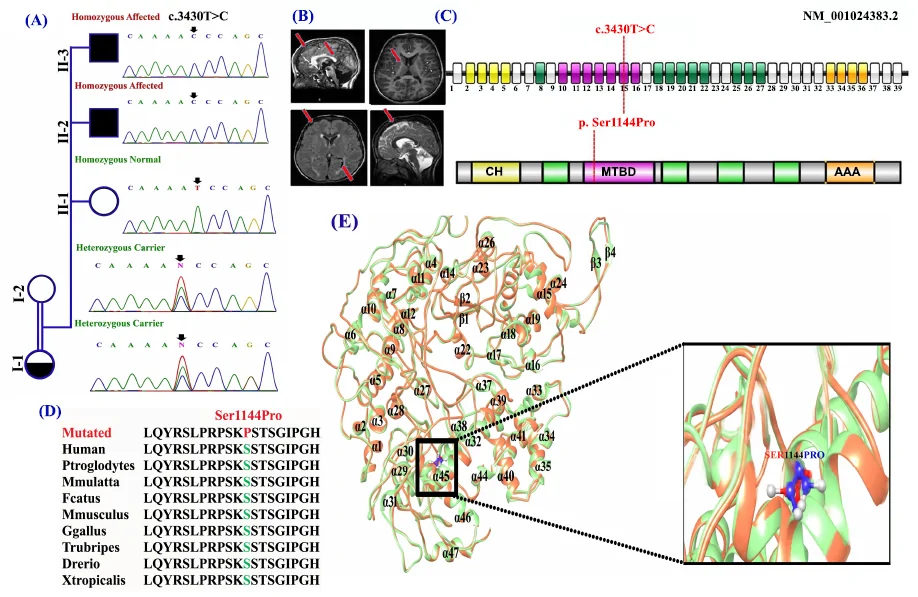
(**A**) Pedigree of the family with autosomal recessive neurodevelopmental disease. Sanger sequencing chromatograms are shown beneath each individual. The arrow represent the point of mutation (c.3430T>C). (**B**) MRI of the affected individual (II-2) showing abnormal brain MRI. The arrow indicated generalized cerebellar atrophy predominantly in ventricles and folia. (**C**) Representation of the gene structure and protein domains of the NAV3. Red line represent the mutation identified in the present study. (**D**) Partial sequencing of the NAV3, showing conservation of the Ser1144 across different species. (**E**) Structural differences along with superimposition of NAV3^WT^ and NAV3^SER1144PRO^ were signifying in coral and light sea green colors. Wild type and mutated residues depicted in red and blue color, respectively. The secondary structures were labeled in black color. The zoom in inset signifying the position of mutation. The effect indicates that α13 is converted into loop, there is induction of α-helix between α18 and β3 and α22 is also converted into loop.

**Fig. (2):**
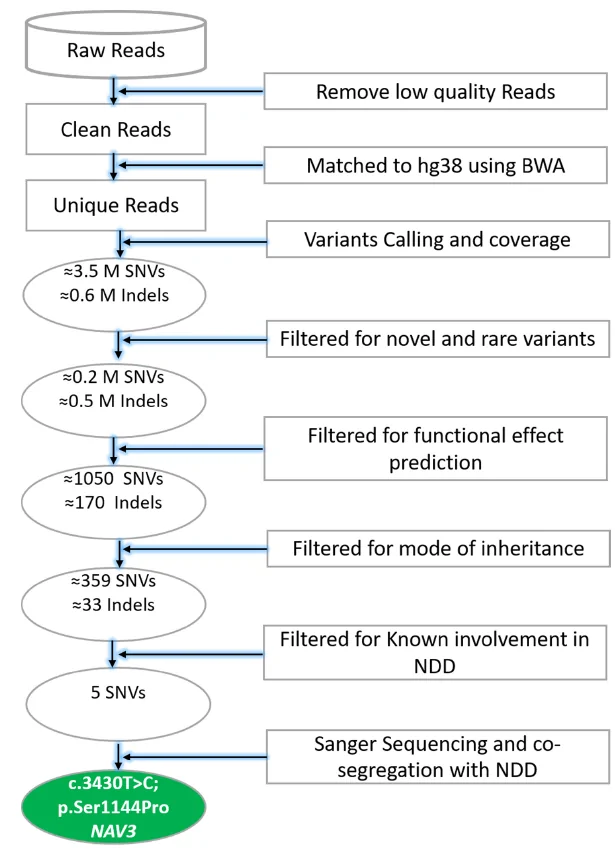
Variant discovery pipeline used for the analysis of whole-genome sequencing data obtained for familial NDD cases.

**Table 1:** Phenotypic characteristics of individuals harboring *NAV3* variants.

**-**	**Family ID**	**Variant**	**Genotype**	**Ethnicity**	**Individual**	**Sex**	**Current Age (Years)**	**Age of Onset**	**Intellectual Disability**	**Speech Delay**	**Developmental Delay**	**Head Abnormalities**	**Structural Abnormalities**	**Behavioral Problems**	**Eye Anomalies**	**Ear Anomalies**	**Heart Defects**	**Neuro/Muscular Issues**	**Congenital Anomalies**	**Other**
1	Ghaffar *et al.*, 2024-PKMR471	p.(Arg2261Cys)	Homozygous	Pakistani	IV:3	Male	22	By birth	Severe	Yes	Yes	Microcephaly	Dentofacial abnormality, curved backbone, hypertelorism, feet deformity	Aggressive	Hypertelorism, mild strabismus	No	No	Cannot walk without support, dystonia	No	Hypotonia
2	Ghaffar *et al.*, 2024-PKMR471	p.(Arg2261Cys)	Homozygous	Pakistani	IV:4	Male	14	By birth	Severe	Yes	Yes	Microcephaly	Dentofacial abnormality, curved backbone, hypertelorism, feet deformity	Aggressive	Hypertelorism, strabismus (Exotropia)	No	No	Cannot walk without support	No	Hypotonia
3	Ghaffar *et al.*, 2024-PE30	p.(Ser1326Asn)	Homozygous	Pakistani	II:4	Female	19	By birth	Moderate	Yes	Yes	Microcephaly	None	Hyperactivity with stereotypic hand movements	No	No	No	NA	Pigmentary skin changes, normal EEG	-
4	Ghaffar *et al.*, 2024-MI01	p.(Ser1681Arg)	De novo	-	II:5	Female	19	By birth	Moderate	Yes	Yes	Microcephaly	None	Hyperactivity with stereotypic hand movements	No	No	No	NA	NA	
5	Ghaffar *et al.*, 2024-F01	p.(Thr1617Tyrfs*9)	Homozygous	Caucasian	II:1	Male	NA	NA	Mild	Yes	Yes	NA	Short hands, hypertelorism, long palpebral fissures	ADHD, frustration	Strabismus (hypermetropia), large eyebrows	Large ear lobules	NA	NA	NA	-
6	Ghaffar *et al.*, 2024-PED4263	p.(Asp1208Argfs*58)	De novo	French	II:1	Male	8	ND	Mild	Yes	Yes	+1.4 SD	Pointed chin	ADHD	No	No	NA	NA	NA	Large, crumbled café-au-lait stain on chest and back
7	Ghaffar *et al.*, 2024-PK1	p.(Trp332*)	Homozygous	Pakistani	II:1	Male	4	By birth	Mild	Yes	Yes	+2 SD	No	Aggressive, introverted	No	No	No	Cannot walk without support, generalized dystonia, wheelchair-bound	Testicular ectopia	Hypotonia
8	Ghaffar *et al.*, 2024-FM1	p.(Trp2270*)	Heterozygous	European	I:1	Female	50	ND	Mild	Yes	Yes	No	No	No	No	No	No	Mild dystonia (face)	ND	Movement disorder (dystonia)
9	Umair *et al.*, 2024	c.2604_2605delAG; p.Val870SerfsTer12	Homozygous	Saudi Arabian	IV:4	Female	13	By birth	Mild	Yes	Yes	Normal	None	Seizure	Normal	Normal	Normal	Normal	Normal reflexes, hypotonia	NA
10	Present study	p.Ser1144Pro	Homozygous	Pakistani	II:2	Male	10	By birth	Moderate	Yes	Yes	Microcephaly	None	febrile seizures	Normal	Normal	Normal	Normal	Normal reflexes, hypotonia	NA
11	Present study	p.Ser1144Pro	Homozygous	Pakistani	II:3	Male	14	By birth	Moderate	Yes	Yes	Microcephaly	None	No	Normal	Normal	Normal	Normal	Normal reflexes, hypotonia	NA

**Table 2:** Genetic variant interpretation.

**Variant**	**Effect**	**Evidence**
NAV3 12:78119626 T > C	Pathogenic (II)	PS1, PS2, PS3, PS4, PP1-S, PM1, PM2, PM3, PM4, PM5, PM6
-	Gene predictive scores	
-	pLI	1.00
-	LOEUF	0.43
-	sHet	0.070
-	pHaplo	0.97
-	pTriplo	0.83
-	Missense Z Score	2.18
-	Protein predictive scores	
-	pDN	0.422
-	pGOF	0.421
-	pLOF	0.785

**Table 3:** To-date variants reported in the *NAV3* gene.

**S/No**	**Consanguinity**	**Phenotype**	**cDNA**	**Protein**	**Type**	**References**
1	Yes	NDD	c.6847C>T	Arg2261Cys	Homozygous	[14]
2	Yes	NDD	c.996G>A	Trop332*	Homozygous	[14]
3	Yes	NDD	c.3977G>A	Ser1326Asn	Homozygous	[14]
4	No	NDD	c.4848_4849 delAA	Thr1617Tyrfs*9	Heterozygous	[14]
5	No	NDD	c.6810G>A	Trp2270*	Heterozygous	[14]
6	No	NDD	c.3621InsA	Asp1208Argfs*58	Heterozygous	[14]
7	No	NDD	c.5043C>G	Spr1681Arg	Heterozygous	[14]
9	Yes	NDD	c.2604_2605delAG;	Val870SerfsTer12	Homozygous	[15]
10	Yes	NDD	c.3430T>C	Ser1144Pro	Homozygous	Present Study

**Table 4:** Pathogenicity of the identified variant [c.3430T>C].

***In silco* Tool**	**Score**	**Prediction**	**Rankscore**
REVEL	0.324, 0.324	VUS	0.6462
BayesDel noAF	noAF score-0.116	Tolerated	noAF rankscore 0.6213
BayesDel addAF	addAF score0.08476	Damaging	addAF rankscore 0.6259
MetaRNN	0.5365, 0.5365, 0.5365	Damaging, Damaging, Damaging	0.6348
FATHMM-XF	coding score0.9568	Damaging	coding rankscore 0.9756
MutPred	0.23, 0.23	VUS	0.157
EIGEN	raw coding0.7489	VUS	raw coding rankscore 0.8284
DEOGEN2	0.3432	Tolerated	0.7119
M-CAP	0.05218	Damaging	0.6497
SIFT	0.072, 0.056	Tolerated, Tolerated	converted rankscore 0.3819
DANN	0.9991	VUS	
Mutation assessor	2.44, 2.44	Medium, Medium	0.7076
LIST-S2	0.9424, 0.9526, 0.9394	Damaging, Damaging, Damaging	0.8193
MutationTaster	1, 1, 1, 1	Disease causing, Disease causing, Disease causing, Disease causing	converted rankscore 0.81
PrimateAI	0.6661	Tolerated	0.6228
PROVEAN	-3.4, -3.33	Damaging, Damaging	converted rankscore 0.6701

## Data Availability

All the data and supporting information are provided within the article.

## LIST OF ABBREVIATIONS

NDDs: Neurodevelopmental Disorders
GDD: Global Developmental Delay
ASD: Autism Spectrum Disorder
WGS: Whole-Genome Sequencing
ID: Intellectual Disability
ADHD: Attention Deficit Hyperactivity Disorder
SNPs: Single-Nucleotide Polymorphisms
WES: Whole-Exome Sequencing
SNVs: Single-Nucleotide Variants
